# C/EBPα is indispensable for PML/RARα-mediated suppression of long non-coding RNA NEAT1 in acute promyelocytic leukemia cells

**DOI:** 10.18632/aging.203000

**Published:** 2021-04-26

**Authors:** Doudou Tang, Piao Hu, Dengqin Zhu, Yujiao Luo, Mingjie Chen, Guangsen Zhang, Yewei Wang

**Affiliations:** 1 Department of Respiratory and Critical Care Medicine, The Second Xiangya Hospital, Hunan Centre for Evidence-Based Medicine, Central South University, Changsha, Hunan, China; 2 Department of Hematology, The Second Xiangya Hospital, Central South University, Changsha, Hunan, China; 3 Institute of Molecular Hematology, Central South University, Changsha, Hunan, China; 4 Cloud-Seq Bio-Tech Inc., Shanghai, China

**Keywords:** PML/RARα, C/EBPα, NEAT1, transcriptional regulation, APL

## Abstract

Better understanding of the transcriptional regulatory network in acute promyelocytic leukemia (APL) cells is critical to illustrate the pathogenesis of other types of acute myeloid leukemia. Previous studies have primarily focused on the retinoic acid signaling pathway and how it is interfered with by promyelocytic leukemia/retinoic acid receptor-α (PML/RARα) fusion protein. However, this hardly explains how APL cells are blocked at the promyelocytic stage. Here, we demonstrated that C/EBPα bound and transactivated the promoter of long non-coding RNA NEAT1, an essential element for terminal differentiation of APL cells, through C/EBP binding sites. More importantly, PML/RARα repressed C/EBPα-mediated transactivation of NEAT1 through binding to NEAT1 promoter. Consistently, mutation of the C/EBP sites or deletion of retinoic acid responsive elements (RAREs) and RARE half motifs abrogated the PML/RARα-mediated repression. Moreover, silencing of C/EBPα attenuated ATRA-induced NEAT1 upregulation and APL cell differentiation. Finally, simultaneous knockdown of C/EBPα and C/EBPβ reduces ATRA-induced upregulation of C/EBPε and dramatically impaired NEAT1 activation and APL cell differentiation. In sum, C/EBPα binds and transactivates NEAT1 whereas PML/RARα represses this process. This study describes an essential role for C/EBPα in PML/RARα-mediated repression of NEAT1 and suggests that PML/RARα could contribute to the pathogenesis of APL through suppressing C/EBPα targets.

## INTRODUCTION

Acute promyelocytic leukemia (APL), a unique subtype of acute myeloid leukemia (AML), is characterized by the specific chromosomal translocation t(15;17)(q22;q21) and promyelocytic leukemia/retinoic acid receptor-α (PML/RARα) fusion protein, which is considered to be the initiating event of APL [1, 2]. In general, PML/RARα acts as a strong transcriptional repressor for its target genes by recruiting corepressor molecules, ultimately resulting in a distinctive differentiation block at the promyelocytic stage [3–5]. All-trans retinoic acid (ATRA) is able to trigger PML/RARα degradation and restore the expression of affected genes, eventually leading to terminal differentiation of APL blasts and disease remission [6]. PML/RARα retains the DNA-binding domain of retinoic acid receptor-α (RARα), which enables the direct repression of classical targets of the retinoic acid signaling pathway [4]. However, interference of RARα-mediated transcription alone hardly affects myeloid lineage commitment [7]. Based on this observation, PML/RARα has been found to interact with other myeloid transcription factors, such as AP-1 [8], Sp1 [9], GATA-2 [10], and PU.1 [11], and target their downstream elements, thus repressing a variety of genes that are essential for granulocytic differentiation and adding additional complexity to its action.

Long non-coding RNA (lncRNA) nuclear enriched abundant transcript 1 (NEAT1) is a recently discovered essential component of nuclear paraspeckles and plays a critical role in the regulation of gene expression [12]. Dysregulation of NEAT1 is associated with several cancers [13]. In APL cells, PML/RARα oncoprotein markedly represses NEAT1 expression whereas ATRA-induced activation of NEAT1 is essential for granulocytic differentiation of APL cells [14]. In a previous study, we demonstrated that ATRA-induced upregulation of NEAT1 required de novo protein synthesis, and C/EBP family transcription factor C/EBPβ directly bound and transactivated the promoter of NEAT1 [15]. However, several questions remain to be answered. First, NEAT1 increased by 4-fold 24 hours after ATRA treatment whereas overexpression of C/EBPβ only resulted in an about 2-fold increase of NEAT1 promoter activity. Second, knockdown of C/EBPβ only slightly impaired ATRA-induced upregulation of NEAT1. Hence, additional factors may contribute to the activation of NEAT1 during APL cell differentiation.

C/EBPs are a family of transcription factors that share common structural and functional properties, and binding sites [16]. C/EBPα, the founding member of the C/EBP family, plays a crucial role in granulopoiesis [17] and C/EBPα knockout mice are deficient in neutrophils and eosinophils [18]. Loss of C/EBPα in myeloid cells leads to a differentiation block *in vitro* and *in vivo*, similar to blasts isolated from AML patients [19]. Moreover, impairment of C/EBPα function partially contributes to the development of APL [20]. In contrast, ectopic expression of C/EBPα can restore differentiation of the leukemic blasts [21, 22], and prolongs survival of APL-bearing mice [23]. Taken together, C/EBPα plays an important role in granulocytic differentiation and may be also involved in NEAT1 upregulation.

In the present study, we found that C/EBPα directly bound to and transactivated the promoter of NEAT1 via the -1453 and -54 C/EBP binding sites. More importantly, PML/RARα bound to the promoter of NEAT1 and repressed the C/EBPα-mediated transactivation whereas mutation of the C/EBP binding sites abrogated the PML/RARα-mediated repression. Furthermore, silencing of C/EBPα attenuated ATRA-induced NEAT1 upregulation and granulocytic differentiation of APL cells. Finally, double knockdown of C/EBPα and C/EBPβ reduces ATRA-induced upregulation of C/EBPε and markedly impaired NEAT1 activation and APL cell differentiation. This study reveals a previously unidentified role for C/EBPα in PML/RARα-mediated repression of NEAT1 in the pathogenesis of APL.

## RESULTS

### C/EBPα directly binds and transactivates the NEAT1 promoter

**Figure 1:**
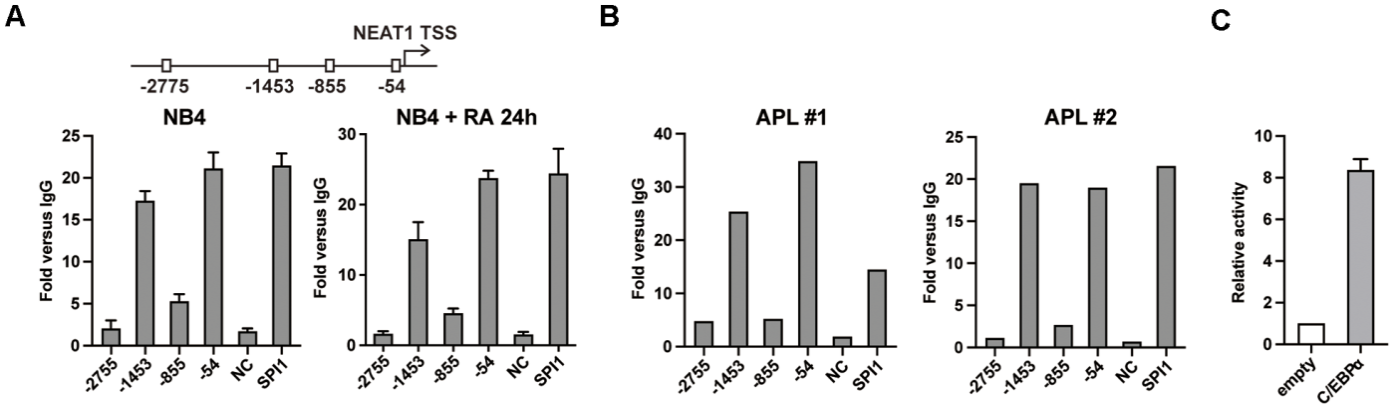
**C/EBPα directly binds and transactivates the promoter region of NEAT1.** (**A**) Upper panel: Schematic representation of putative C/EBP binding sites in the NEAT1 promoter. Lower panel: C/EBPα ChIP-qPCR showing the enrichment of C/EBPα in each putative binding site, the negative control and positive control (*SPI1* promoter) in NB4 cells that were untreated or treated with ATRA at 1μM for 24 h (RA 24h). (**B**) ChIP was performed on two APL patient samples with anti-C/EBPα antibody. DNA fragments at NEAT1 promoter were subsequently measured with qPCR. (**C**) The 1656 bp NEAT1 promoter reporter construct (125 ng) was transfected into 293T cells along with pcDNA3.1 vector (empty) or pcDNA3.1-C/EBPα (C/EBPα) expression plasmid (500 ng). The data represent the mean ± S.E.M from 3 replicates.

Previously, we found that C/EBP family member C/EBPβ directly bound to and transactivated NEAT1 promoter via C/EBP binding sites [15]. However, overexpression of C/EBPβ only resulted in slight activation of NEAT1. Based on the crucial role of C/EBPα in granulopoiesis [17], we hypothesize that it contributes to the regulation of NEAT1 in a similar pattern to C/EBPβ. As shown in Figure 1A, the chromatin immunoprecipitation (ChIP)-qPCR results showed that the regions around -1453 bp and -54 bp sites were obviously precipitated with anti-C/EBPα antibody in both untreated and ATRA-treated NB4 cells. The findings were validated on samples isolated from APL patients (Figure 1B). Then in the luciferase reporter assays with 293T cells which do not express endogenous C/EBPα, 1656 bp NEAT1 promoter construct encompassing the -1453 bp and -54 bp sites was activated by C/EBPα (Figure 1C). The above findings suggest that C/EBPα can bind to and transactivate the promoter of NEAT1 directly.

### C/EBPα activates the NEAT1 promoter through both the -1453 and -54 C/EBP binding sites

**Figure 2:**
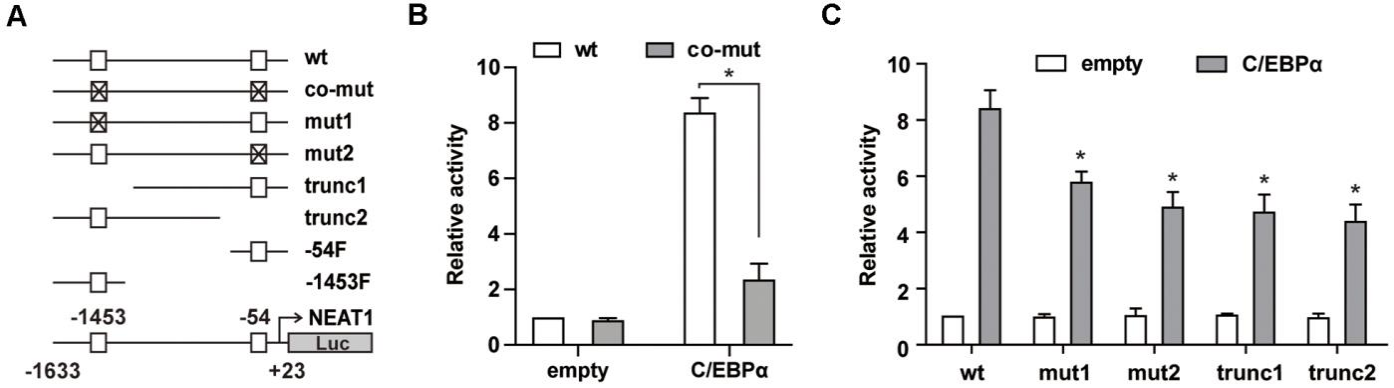
**C/EBPα transactivates NEAT1 through the -1453 and -54 sites in the NEAT1 promoter.** (**A**) Schema of the NEAT1 promoter region shows the different mutation/truncation constructs used in this study. □ represents the wild-type C/EBP binding site and ⊠ represents the mutated site. (**B**) The wild-type (wt) or double mutated (co-mut) promoter construct (125 ng) was co-transfected into 293T cells along with the C/EBPα expression construct (500 ng). (**C**) Different mutation/truncation luciferase promoter plasmids were co-transfected with 500ng of the pcDNA3.1 (empty) or pcDNA3.1-C/EBPα (C/EBPα) vector into 293T cells. The data represent the mean ± S.E.M from three replicates. * indicates *p*<0.05.

We further used a series of mutated and truncated NEAT1 promoter reporters constructed previously [15] (Figure 2A) to test the importance of -1453 and -54 sites in C/EBPα-mediated transactivation. As shown in Figure 2B, the luciferase assay results showed that double mutation of -1453 and -54 sites (co-mut) significantly impaired C/EBPα-mediated transactivation. Then we used the -1453 or -54 C/EBP site single mutated construct and 5’ or 3’ truncation of the NEAT1 promoter to perform the luciferase assay. The promoter activity of either site (-1453 or -54) mutated or truncated constructs was markedly attenuated (Figure 2C), indicating that C/EBPα transactivates the NEAT1 promoter through both -1453 and -54 sites.

### PML/RARα represses the C/EBPα-mediated transactivation of NEAT1 through binding to NEAT1 promoter

**Figure 3:**
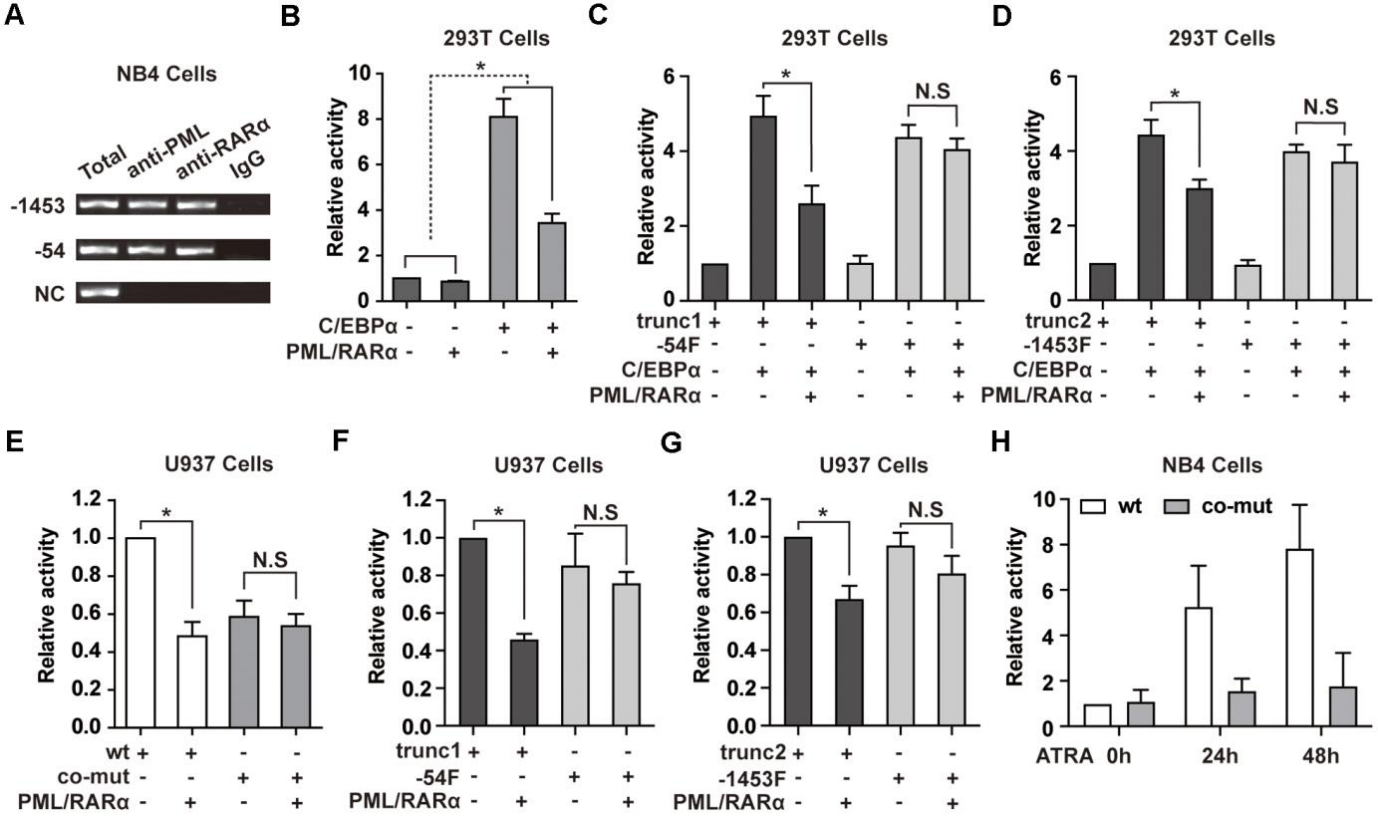
**PML/RARα binds to NEAT1 promoter and represses the C/EBPα-mediated transactivation of NEAT1.** (**A**) ChIP was performed in NB4 cells with anti-PML, anti-RARα, or nonspecific (normal immunoglobulin G (IgG)) antibodies. The immunoprecipitated DNA was amplified by PCR, followed by agarose electrophoresis. (**B**) The promoter of NEAT1 was co-transfected into 293T cells along with pcDNA3.1 vector or pcDNA3.1-PML/RARα expression plasmid in the absence or presence of C/EBPα. (-) and (+) represent the absence or presence of the indicated plasmid. (**C**, **D**) NEAT1 promoter truncation plasmids that contain (trunc1 and trunc2) or do not contain RARE and RARE half motifs (-54F and -1453F) were co-transfected with pcDNA3.1 vector or pcDNA3.1-C/EBPα and with or without PML/RARα-expression construct. Luciferase activity was detected 24 h after transfection. (**E**) The wild-type (wt) or double mutated (co-mut) NEAT1 promoter construct was co-transfected into U937 cells along with pcDNA3.1 vector or pcDNA3.1-PML/RARα expression plasmid. (**F**, **G**) NEAT1 promoter truncation plasmids in the presence (trunc1 and trunc2) or absence of RARE and RARE half motifs (-54F and -1453F) were co-transfected with pcDNA3.1 vector or pcDNA3.1-PML/RARα expression construct. (**H**) The wild-type (wt) or double mutated (co-mut) NEAT1 promoter construct was transfected into NB4 cells. Six hours later, cells were treated with ATRA and tested at the indicated time points. The error bar represents the standard error of the mean (S.E.M.) (n=3). * indicates *p*<0.05.

PML/RARα is able to repress its target genes directly and is also capable to interact with myeloid transcription factors to suppress their target genes [4, 11]. Motif scanning of NEAT1 promoter using AMD tool [24] revealed enrichment of potential retinoic acid responsive elements (RAREs) and RARE half motifs near the -1453 and -54 sites (Supplementary Tables 1, 2). We first tested whether PML/RARα could bind to the promoter region of NEAT1. ChIP assays were performed in NB4 cells. As shown in Figure 3A, PML/RARα bound to the -1453 and -54 regions of the NEAT1 promoter. The results were further validated in bone marrow cells from two APL patients (Supplementary Figure 1). Then we investigated whether PML/RARα represses the NEAT1 promoter directly, luciferase reporter assays were conducted in 293T cells. As shown in Figure 3B, transfection of PML/RARα alone resulted in a minimal decrease of NEAT1 promoter activity. Interestingly, C/EBPα-mediated transactivation of NEAT1 was markedly suppressed by PML/RARα (Figure 3B), suggesting that the repression effect of PML/RARα is specific to C/EBPα-mediated transcriptional activation of NEAT1 promoter. Because there are several potential RAREs and RARE half motifs near the -1453 and -54 sites (Supplementary Tables 1, 2), it is difficult to mutate all the RAREs and RARE half motifs on the 1656 bp NEAT1 promoter construct. Therefore, we used truncations around -54 and -1453 sites (-54F and -1453F), which do not contain the potential RAREs and RARE half motifs, to further elucidate whether PML/RARα inhibits C/EBPα-mediated transactivation through direct binding to NEAT1 promoter. As shown in Figure 3C, C/EBPα markedly enhanced the promoter activity of the trunc1 construct, which contains the -54 C/EBP site and potential RAREs and RARE half motifs, whereas PML/RARα significantly suppressed this effect. In contrast, though C/EBPα significantly activated the -54F construct, PML/RARα could not significantly repress the C/EBPα-mediated transactivation. Similar results were also found in the trunc2 construct and -1453F construct (Figure 3D). All these results indicated that direct binding of PML/RARα to NEAT1 promoter is required for its repression of C/EBPα-mediated transactivation.

To further elucidate whether C/EBPα is required for PML/RARα-mediated repression of NEAT1 in hematopoietic cells, PML/RARα expression plasmid was co-transfected along with the wild-type NEAT1 promoter or the C/EBP sites double mutated construct in U937 cells, which endogenously express C/EBPα. As illustrated in Figure 3E, PML/RARα-mediated repression of NEAT1 was abolished in the co-mut promoter construct. Together with Figure 3B, the results indicated that PML/RARα functioned as an effective repressor of NEAT1 only in the presence of C/EBPα. Then we used the truncations around the -54 or -1453 site to test the repression effect of PML/RARα on NEAT1 promoter in U937 cells. As shown in Figure 3F, 3G, PML/RARα effectively suppressed the promoter activity of trunc1 and trunc2 constructs, which contains potential RAREs and RARE half motifs. Unsurprisingly when using the truncations without the potential RAREs and RARE half motifs, -54F and -1453F, PML/RARα could not significantly repress the NEAT1 promoter activity. These results collectively suggest that PML/RARα represses C/EBPα-mediated transactivation of NEAT1 through binding to NEAT1 promoter and C/EBPα is indispensable for PML/RARα-mediated suppression of NEAT1.

Additionally, the luciferase reporter assays were performed in NB4 cells to clarify the responsiveness of the NEAT1 promoter to ATRA. As demonstrated in Figure 3H, the luciferase activity of the NEAT1 promoter was dramatically increased after ATRA treatment. However, there was no obvious response of co-mut construct to ATRA treatment. This result is in accordance with the upregulation of NEAT1 after ATRA treatment in NB4 cells and indicates that suppression of C/EBPα-mediated transactivation of NEAT1 by PML/RARα is relieved by ATRA.

### Knockdown of C/EBPα attenuates ATRA-induced NEAT1 upregulation and APL cell differentiation

**Figure 4:**
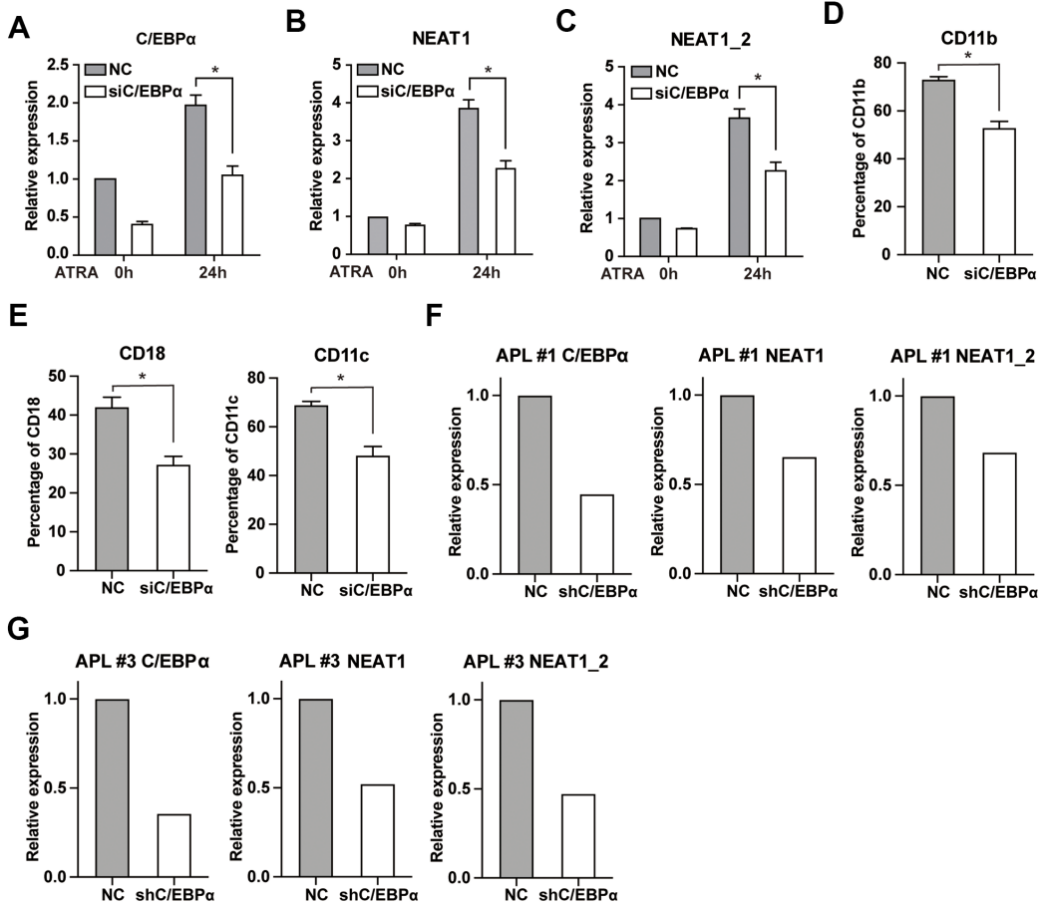
**Knockdown of C/EBPα attenuates ATRA-induced upregulation of NEAT1 and NB4 cell differentiation.** (**A**) NB4 cells were transfected with 3 μg siRNA targeting C/EBPα (siC/EBPα) or negative control siRNA (NC). Six hours later, cells were treated with 1μM ATRA for 24 h. Expression of C/EBPα was subsequently determined by qRT-PCR. (**B**, **C**) Expression of NEAT1 and NEAT1_2 isoform in C/EBPα-silenced NB4 cells was detected both before and after ATRA treatment. (**D**, **E**) The granulocytic differentiation marker CD11b, CD18, and CD11c in C/EBPα-silenced NB4 cells were tested after ATRA treatment for 24 h. The data represent the mean ± S.E.M from three replicates. * indicates *p*<0.05. (**F**, **G**) The expression of NEAT1 and NEAT1_2 isoform in C/EBPα-silenced primary APL bone marrow cells was measured after ATRA treatment for 24 h.

Next, we sought to determine the effect of C/EBPα on ATRA-induced upregulation of NEAT1. We silenced C/EBPα expression via siRNA in NB4 cells. The reduction of C/EBPα was confirmed by qRT-PCR (Figure 4A). As shown in Figure 4B, 4C, knockdown of C/EBPα resulted in obviously decreased expression of NEAT1 and NEAT1_2 isoform after ATRA treatment. In the meantime, silencing of C/EBPα led to a significant decrease in ATRA-induce differentiation of NB4 cells (Figure 4D, 4E). These findings indicate that C/EBPα is required for full induction of NEAT1 by ATRA. Then the results were further confirmed in bone marrow cells from two APL patients (Figure 4F, 4G).

### Double knockdown of C/EBPα and C/EBPβ reduces ATRA-induced upregulation of C/EBPε and dramatically impairs NEAT1 activation and APL cell differentiation

C/EBPα and C/EBPβ regulate a number of myeloid lineage-specific genes. For example, conditional expression of C/EBPα in U937 and HL-60 cells upregulates C/EBPε [16, 25]. Similarly, ATRA-induced activation of C/EBPβ in APL cells induces expression of C/EBPε [26]. Therefore, we hypothesize that C/EBPα and C/EBPβ may act upstream of C/EBPε and play a more critical role during APL cell differentiation.

**Figure 5:**
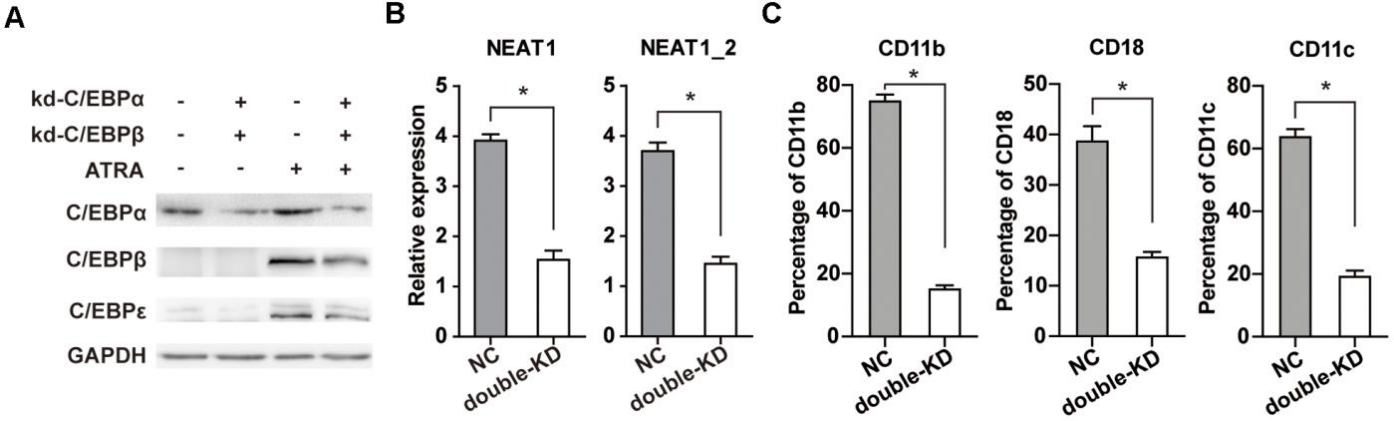
**Double knockdown of C/EBPα and C/EBPβ reduces ATRA-induced upregulation of C/EBPε and markedly impairs NEAT1 upregulation and NB4 cell differentiation.** (**A**) C/EBPβ knockdown (kd-C/EBPβ) or control (NC) NB4 cells were transfected with C/EBPα siRNA (kd-C/EBPα) or negative control siRNA (NC). The protein levels of C/EBPα, C/EBPβ, C/EBPε, and GAPDH were determined in NB4 cells before and after ATRA treatment (1 μM for 24 h). (**B**) Expression of NEAT1 and NEAT1_2 isoform in C/EBPα and C/EBPβ double-silenced (double-KD) NB4 cells was analyzed after ATRA treatment for 24 h. (**C**) Flow cytometric analysis of CD11b, CD18, and CD11c expression in NB4 cells with or without C/EBPα and C/EBPβ double knockdown (double-KD) following ATRA treatment for 24h. The data represent the mean ± S.E.M. from three replicates. * indicates *p*<0.05.

We generated NB4 cells that stably express shRNA targeted C/EBPβ (kd-C/EBPβ) or negative control shRNA (NC) previously [15], and introduced a specific siRNA to silence C/EBPα. The protein levels of C/EBPα, C/EBPβ, and C/EBPε were determined both before and after ATRA treatment. As shown in Figure 5A, ATRA upregulated C/EBPα, C/EBPβ, and C/EBPε, whereas simultaneous knockdown of C/EBPα and C/EBPβ reduces ATRA-induced upregulation of C/EBPε, suggesting that C/EBPα and C/EBPβ are upstream regulators during APL cell differentiation. Consistently, simultaneous knockdown of C/EBPα and C/EBPβ markedly attenuated ATRA-induced NEAT1 upregulation (Figure 5B) and granulocytic differentiation in NB4 cells (Figure 5C). Taken together, our results demonstrate that double knockdown of C/EBPα and C/EBPβ, not only decreases C/EBPε upregulation, but also markedly attenuates ATRA-induced NEAT1 upregulation and APL cell differentiation.

## DISCUSSION

C/EBPs are a family of transcription factors that regulate cell growth and differentiation. As the founding members of this family, C/EBPα, is a key transcriptional regulator of granulopoiesis. PML/RARα is the initiating event of APL which interferes with its target genes through multiple ways. In this study, we demonstrate an important role for C/EBPα in activating the expression of lncRNA NEAT1. More importantly, PML/RARα represses the C/EBPα-mediated transactivation through binding to the NEAT promoter whereas mutation of the C/EBP sites abrogates this effect. Our results shed light on the transcriptional regulation of lncRNAs and the role of C/EBPα in mediating the PML/RARα-dependent transcriptional repression during APL pathogenesis.

Previously, we reported that C/EBPβ bound to and transactivated NEAT1 [15]. C/EBPα, another member of C/EBP family transcription factor, plays a critical role in granulocytic differentiation [17, 27] through targets and activates several key hematopoietic genes, including *G-CSF* receptor [28], *C/EBPε* [16], *SPI1* [16], *MPO* [28], and *ELANE* [29]. In this study, we found that C/EBPα transactivated NEAT1 through the same C/EBP binding sites as C/EBPβ. Furthermore, C/EBPα is more efficient than C/EBPβ in transactivating the NEAT1 promoter. In addition, a combination knockdown of C/EBPα and C/EBPβ reduced ATRA-induced upregulation of C/EBPε in APL cells. These results suggest that C/EBPα may be the major activator of NEAT1 in APL.

PML/RARα is reported to repress the expression of NEAT1 in U937-PR9 cells [14]. We previously found that NEAT1 was not a direct ATRA-responsive gene [15], and here we reveal that PML/RARα binds to the NEAT1 promoter and repressed C/EBPα-mediated transactivation. PML/RARα retains the protein-protein interaction domain of PML, thus it is able to directly interact with many hematopoietic transcription factors and affect their target genes [11]. For example, PML/RARα is found to repress AP-1-dependent transactivation, which can be reversed by ATRA [8]. PML/RARα can also physically associate with GATA-2 and influence GATA2-dependent gene transcription [10]. Similarly, PML/RARα is reported to bind to and target the promoter regions that contain both PU.1 and RARE half sites and has been bound by PU.1 [11]. On the other hand, C/EBPα is capable of interacting with other transcription factors and proteins apart from dimerizing with members of the C/EBP family [30]. For instance, C/EBPα physically interacts with E2F to inhibit its transactivation activity, ultimately contributing to myeloid differentiation [31]. C/EBPα also directly interacts with CDK2 and CDK4 and blocks the association of CDK2 and CDK4 with cyclins, leading to cell growth arrest [32]. In addition, C/EBPα directly interacts and cooperates with p21 to inhibit CDK2 activity [33]. In hematopoietic cell lines, C/EBPα activates BCL-2 by directly interacting with NF-κBp50, thus inhibiting apoptosis, which may contribute to leukemogenesis [34]. AML1-ETO fusion protein, the most common chimeric protein in AML, is able to physically interact with C/EBPα and suppress C/EBPα-dependent activation [35, 36]. These results collectively raise the possibility that PML/RARα may interact directly with C/EBPα and repress C/EBPα-mediated transactivation in the pathogenesis of APL. It has been reported that conditional induction of PML/RARα in myeloid U937-PR9 cells decreases C/EBPα expression at both mRNA and protein levels [37]. In clinical samples, a report revealed that expression of C/EBPα in APL is lower than that of normal bone marrow [38], whereas others found that there were no significant differences in C/EBPα expression between APL and normal bone marrow samples [35, 39]. In line with the previous reports, we found that there was a considerable expression of C/EBPα in NB4 cells. Both results suggest that PML/RARα could not completely inhibit the transcription of C/EBPα, raising the possibility that PML/RARα may repress the function of C/EBPα protein. In our results, despite direct binding of PML/RARα to NEAT1 promoter, PML/RARα did not significantly suppress the promoter activity of NEAT1 in absence of C/EBPα. Taken together, we propose that PML/RARα contributes to the pathogenesis of APL, not only through suppression of C/EBPα itself but also, at least in part, through repression of C/EBPα targets, such as NEAT1.

Furthermore, C/EBPα and C/EBPβ play different roles even both could bind to and transactivate NEAT1 in APL cells. Based on the finding that C/EBPα is a critical factor during the transition from myeloblast to promyelocyte [16], we speculate that C/EBPα may initially bind to the NEAT1 promoter prior to promyelocyte stage. The binding of C/EBPα was repressed by PML/RARα, which may contribute to the pathogenesis of APL. In an ATRA-induced NB4 cell granulocytic differentiation model, the binding of C/EBPα to the G-CSF promoter remains stable within 24 hours and disappears after 48 hours of ATRA treatment [26]. Consistently, we found that the binding of C/EBPα in the NEAT1 promoter did not reduce after ATRA treatment for 24 hours. Restoring C/EBPα transactivation on its targets by ATRA-induced degradation and/or dissociation of PML/RARα, may be involved in APL cell differentiation. On the contrary, C/EBPβ was hardly detectable at both RNA and protein levels in untreated NB4 cells [26]. However, expression of C/EBPβ was drastically increased following ATRA treatment and the upregulation was in line with the progression of granulocytic differentiation [26, 40]. In ATRA-treated NB4 cells, increased C/EBPβ binds to and activates NEAT1 thereby participating in APL cell differentiation [15].

In conclusion, C/EBPα binds to and transactivates NEAT1, which is repressed by PML/RARα, whereas lack of C/EBPα abrogates this repression. Our results indicate that C/EBPα is required for PML/RARα-mediated repression of NEAT1 in APL. The findings reveal an essential role of C/EBPα in mediating the repression of PML/RARα on its targets and shed light on the potential role of C/EBPα in the regulation of lncRNAs as well. The interaction of PML/RARα with C/EBPα and other transcription factors enables the formation of a broader spectrum of target genes and a cascade gain of function for this fusion protein during the pathogenesis of APL.

## MATERIALS AND METHODS

### Cell culture and reagent

NB4 and U937 cells were cultured in RPMI 1640 medium (Gibco, Carlsbad, CA, USA) containing 10% fetal bovine serum (FBS) (Gibco). The 293T cells were maintained in DMEM (Gibco) supplemented with 10% FBS. Cells were grown in a humidified atmosphere with 5% CO_2_ and at 37° C. All-trans retinoic acid (Sigma-Aldrich, St. Louis, MO, USA) was used at a final concentration of 1 μM.

### Patient samples

This study was approved by the institutional review board of the Second Xiangya Hospital, Central South University and was performed in accordance with the Declaration of Helsinki. Informed consent was obtained from all patients. Bone marrow samples were obtained from 4 patients with de novo APL, and leukemic cells were isolated and cultured as previously described [41]. Patients characteristics were summarized in Supplementary Table 3.

### Quantitative real-time RT-PCR

RNA was extracted using RNAiso plus (TaKaRa, Dalian, Liaoning, China) and reverse transcription was performed with PrimeScript RT reagent Kit (TaKaRa) as described previously [15]. Quantitative real-time PCR (qRT-PCR) was performed in Roche LightCycler 96 system using the SYBR Premix Ex Taq II (TaKaRa). GAPDH was used as an internal control. All primers for quantitative real-time RT-PCR are listed in Supplementary Table 4.

### Chromatin immunoprecipitation assay

Chromatin immunoprecipitation (ChIP) was performed with Pierce Agarose ChIP Kit according to the manufacturer’s instruction (ThermoFisher Scientific, Rockford, IL, USA). The following antibodies were used: C/EBPα (Santa Cruz Biotech, sc-61x), PML (Santa Cruz Biotech, H-238x), RARα (Santa Cruz Biotech, C-20x), and rabbit IgG (Abcam, Cambridge, UK, ab46540). The immunoprecipitated DNA was analyzed by qPCR or amplified by PCR, followed by agarose electrophoresis. All primers for ChIP-qPCR and ChIP-PCR are used as described previously [15].

### Plasmid constructions and site-directed mutagenesis

The wild type 1656 bp NEAT1 promoter and a series of truncated and mutated luciferase reporter plasmid were constructed previously [15]. The C/EBPα sequence was amplified using NB4 cDNA and then cloned to the pcDNA3.1 (+) vector. Detailed primer information is listed in Supplementary Table 5.

### Transient transfection and luciferase reporter assay

U937 and NB4 cells were electro-transfected using the Amaxa Nucleofector II device (Lonza, Cologne, Germany) with Nucleofector Kit V (Lonza); 293T cells were transfected with Lipofectamine 2000 (Invitrogen, Carlsbad, CA, USA) according to the manufacturer’s instructions. The detailed procedure was described previously [15]. Luciferase activity was measured with a luminometer using Dual-Luciferase Reporter Assay System reagents (Promega, Madison, WI, USA) 24 h after transfection (NB4 cells were also measured at 48 h). The renilla luciferase plasmid pRL-SV40 was used as a control for transfection efficiency.

### RNA interference experiment

The siRNA sequence used for C/EBPα knockdown was previously described [41]. The sequence 5’-AGC GUG UAG CUA GCA GAG G-3’ was used as negative control. A total of 2 × 10^6^ NB4 cells stably expressing shRNA targeting C/EBPβ or negative control shRNA (NC) were transfected with 3 μg siRNA as described previously [42]. Lentiviral plasmids expressing short hairpins against C/EBPα (shC/EBPα) or negative control (NC) were constructed using pLVX-shRNA2 vector (Clontech Laboratories, Mountainview, CA, USA) with the same sequence as siRNA (The primers for plasmid construction are listed in Supplementary Table 5). Lentiviral particles were produced by co-transfection of lentiviral plasmids in 293T cells with packaging plasmids pMD2.G and psPAX2, and the supernatant was harvested 48 h afterward. Cells from APL patient samples were transduced (overnight incubation) in the presence of 8 μg/ml of polybrene and subsequently treated with 1 μM ATRA for another 24 h.

### Western blot

Total protein extracts were prepared and western blot was performed as previously described [43]. The following antibodies were used: C/EBPα (Cell Signaling Tech, #2295), C/EBPβ (Santa Cruz Biotech, sc-7962x), C/EBPε (Santa Cruz Biotech, sc-158) and GAPDH (Proteintech, 10494-1-AP).

### Flow cytometry

To determine granulocytic differentiation, NB4 cells were stained with anti-human CD11b, CD11c, and CD18 antibodies (BD Biosciences, San Jose, CA, USA), and processed on a BD FACS Canto II flow cytometer (BD Biosciences, San Jose, CA, USA).

### Statistical analysis

The data were analyzed with Student’s t-test and presented as mean ± S.E.M. Data were obtained from at least three independent experiments. A *p* value of less than 0.05 was considered to be statistically significant (* indicates *p*<0.05).

## Supplementary Material

Supplementary Figure 1

Supplementary Tables
