# Supplementary material for: C/EBPα is indispensable for PML/RARα-mediated suppression of long non-coding RNA NEAT1 in acute promyelocytic leukemia cells
Source: Aging (Albany NY). 2021 Apr 26;13(9):13179–94. doi: 10.18632/aging.203000 (PMC8148485; doi:10.18632/aging.203000)
Supplement: Supplementary Figure 1 [file aging-13-203000-s001.pdf]

SUPPLEMENTARY FIGURE

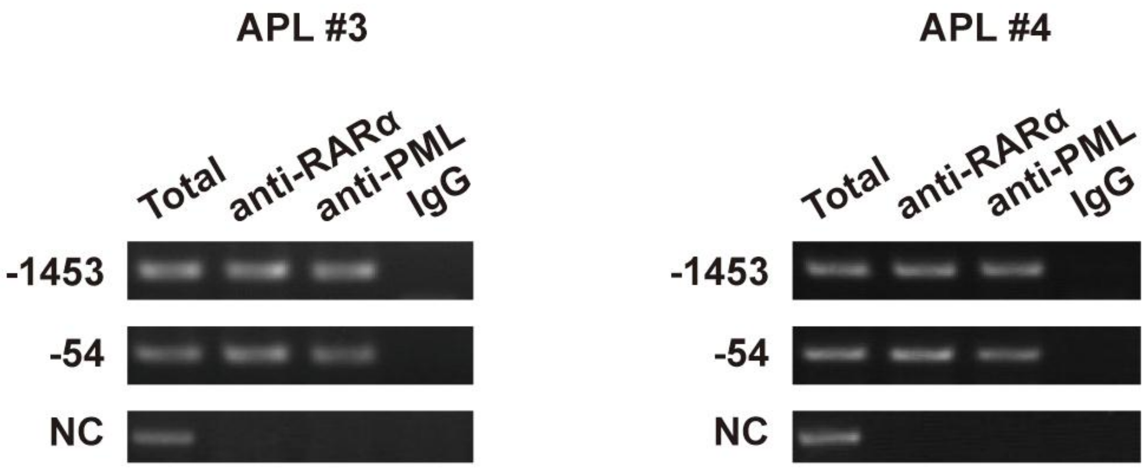

Supplementary Figure 1. ChIP assays were conducted in bone marrow cells from two APL patients with anti-PML, anti-RARα, or nonspecific (IgG) antibodies. Total input and immunoprecipitated DNA were analyzed by PCR using primers around -1453 and -54 sites.
