# Supplementary material for: C/EBPα is indispensable for PML/RARα-mediated suppression of long non-coding RNA NEAT1 in acute promyelocytic leukemia cells
Source: Aging (Albany NY). 2021 Apr 26;13(9):13179–94. doi: 10.18632/aging.203000 (PMC8148485; doi:10.18632/aging.203000)
Supplement: Supplementary Tables [file aging-13-203000-s002.pdf]

SUPPLEMENTARY TABLES

Supplementary Table 1. Predicted retinoic acid responsive elements (RAREs) nearby the -1453 and -54 sites.

Motif	Sequence name	Strand	Start	End	p-value	Matched sequence
V_DR11	neat1promoter	-	80	102	0.0000165	AGGTCAGGAGGCAGGAGAGGGAA
V_DR6	neat1promoter	-	85	102	0.0000418	AGGTCAGGAGGCAGGAGA
V_DR1	neat1promoter	-	90	102	0.0000465	AGGTCAGGAGGCA
V_DR1	neat1promoter	-	97	109	0.00000522	GGGTCCCAGGTCA
V_DR10	neat1promoter	-	97	118	0.0000395	AGCTCTGTGGGGTCCCAGGTCA
V_DR1	neat1promoter	-	150	162	0.0000102	AGTTCCCAGGTCA
V_DR12	neat1promoter	+	231	254	0.0000296	AGTGCAGAGTCACGTGCCAGGGCA
V_DR6	neat1promoter	+	237	254	0.0000406	GAGTCACGTGCCAGGGCA
V_DR9	neat1promoter	+	249	269	0.000034	AGGGCACAGCAATCCCGGGCA
V_DR5	neat1promoter	-	339	355	0.0000906	AGGTCACCACTCAGCCA
V_DR13	neat1promoter	-	350	374	0.0000421	CCTTCAGGCCTCTGCCCTAAGGTCA
V_DR11	neat1promoter	+	509	531	0.000027	TGGTCACATGAGAGGTGGGGGCA
V_DR1	neat1promoter	-	###	1025	0.0000781	GGGTCACTGGTCT
V_DR10	neat1promoter	-	###	1041	0.0000959	CGGCTATTGCCAATCAGGGTCA
V_DR4	neat1promoter	+	###	1121	0.000048	AGGTCAAGGCAGGTGG
V_DR13	neat1promoter	+	###	1135	0.0000205	AAGGCAGGTGGATCACTTGAGGTCA
V_DR4	neat1promoter	+	###	1135	0.00000612	GGATCACTTGAGGTCA
V_DR10	neat1promoter	-	###	1852	0.0000529	AGGGCACTGTTTCAGAGGGTCC
V_DR2	neat1promoter	-	###	1860	0.0000216	AGGACAAAAGGGCA
V_DR13	neat1promoter	+	###	2073	0.0000999	AGTGCAGTGGCACAATCATAGCTCA
V_DR2	neat1promoter	-	###	2240	0.0000824	AGGCCAGGAGTTCG
V_DR9	neat1promoter	+	###	2784	0.0000132	AGGTCAGATGACACACAGTCA
V_DR8	neat1promoter	+	###	2783	0.000045	AGGTCAGATGACACACAGTC
V_DR1	neat1promoter	+	###	2776	0.0000547	AGGTCAGATGACA
V_DR4	neat1promoter	+	###	3152	0.000084	AGGGCTCAGGAGTTCA
V_DR2	neat1promoter	+	###	3152	0.0000285	GGCTCAGGAGTTCA
V_DR2	neat1promoter	+	###	3160	0.0000886	AGTTCACCAGGTTT
V_DR13	neat1promoter	-	###	3814	0.000012	AGGTCACCACGCCCAGCCGAGGCCC
V_DR3	neat1promoter	-	###	3814	0.0000886	AGGTCACCACGCCCA
V_DR4	neat1promoter	-	###	3824	0.00000555	GGGTCGCTTGAGGTCA
V_DR8	neat1promoter	-	###	4150	0.0000508	GGGTCATTGCTCAACGGGAC
V_DR1	neat1promoter	-	###	4150	0.0000816	GGGTCATTGCTCA
V_DR3	neat1promoter	-	###	4159	0.000015	GCGTCACCGGGGTCA

Supplementary Table 2. Predicted retinoic acid responsive element half (RAREhalf) sites nearby the -1453 and -54 sites.

Motif	Sequence name	Strand	Start	End	p-value	Matched sequence
1	neat1promoter	-	97	102	0.000237	AGGTCA
1	neat1promoter	-	150	155	0.000237	AGGTCA
1	neat1promoter	-	214	219	0.000237	AGGTCA
1	neat1promoter	-	350	355	0.000237	AGGTCA
1	neat1promoter	-	970	975	0.000237	AGGTCA
1	neat1promoter	-	3809	3814	0.000237	AGGTCA
1	neat1promoter	+	1106	1111	0.000237	AGGTCA
1	neat1promoter	+	1130	1135	0.000237	AGGTCA
1	neat1promoter	+	2764	2769	0.000237	AGGTCA
1	neat1promoter	-	1020	1025	0.000432	GGGTCA
1	neat1promoter	-	4145	4150	0.000432	GGGTCA
1	neat1promoter	+	3147	3152	0.000915	AGTTCA

Supplementary Table 3. Detailed information about APL patients.

Patient No.	Age at diagnosis (years)	Sex	Peripheral blood counts at diagnosis			Blasts in BM(%)	Cytogenetics	Molecular markers
			WBC ($\times 10^9$/L)	HB (g/L)	PLT ($\times 10^9$/L)			
1	64	F	0.99	53	23	81	46, XX, t(15;17)(q22;q21)[20]	PML-RARa(+)
2	21	F	19.56	105	55	93	46, XX, t(15;17)[15]/46, XX[5]	PML-RARa(+)
3	48	M	1.91	71	16	94.5	46, XY[20]	PML-RARa(+)
4	15	M	5.16	87	31	91	46, XY, t(15;17)(q22;q21)[3]	PML-RARa(+)

Supplementary Table 4. Primers for RT-PCR and quantitative real-time RT-PCR.

	Sequence(5'->3')	Amplicon length
NEAT1-F	CTTCCTCCCTTTAACTTATCCATTCAC	116bp
NEAT1-R	CTCTTCCTCCACCATTACCAACAATAC	
NEAT1_2-F	CAGTTAGTTTATCAGTTCTCCCATCCA	139bp
NEAT1_2-R	GTTGTTGTCGTCACCTTTCAACTCT	
C/EBPα-F	ACGATCAGTCCATCCCAGAG	122bp
C/EBPα-R	TTCACATTGCACAAGGCACT	
GAPDH-F	GGAGCGAGATCCCTCCAAAAT	197bp
GAPDH-R	GGCTGTTGTCATACTTCTCATGG	

Supplementary Table 5. Primers for plasmid construction.

	Sequence(5'->3')
For wt	
NEAT PRO 1633-pGL3-F	CGGGGTACCTTCCCTCTTTCCACACGGTTCT
NEAT PRO 1633-pGL3-R	CCGCTCGAGCATCCCTCCCTGTCGCTAACTC
For trunc1	
NEAT PRO 1368-pGL3-F	CGGGGTACCTGCCTGCTGATACCACCTCAC
NEAT PRO 1633-pGL3-R	CCGCTCGAGCATCCCTCCCTGTCGCTAACTC
For trunc2	
NEAT PRO 1633-pGL3-F	CGGGGTACCTTCCCTCTTTCCACACGGTTCT
NEAT PRO 243-pGL3-R	CCGCTCGAGGCGAATGCCATGAGGAAGAAGA
For mut1	
1453 mut-sense	5'-CAGCACAGAAGGTGGTGATGTGGGTCGCCCAGGCTTGCTC
1453 mut-antisense	5'-GAGCAAGCCTGGGCGACCCACATCACCACCTTCTGTGCTG
For mut2	
54 mut-sense	5'-GTGGAGGAATCGTCCCGTTGAGGTCTGACCCCGGTGACGC
54 mut-antisense	5'-GCGTCACCGGGGTCAGACCTCAACGGGACGATTCCTCCAC
For -54F	
-54F-F	CGGGGTACCTGGAGGAATCGTCCCGTTGAG
NEAT PRO 1633-pGL3-R	CCGCTCGAGCATCCCTCCCTGTCGCTAACTC
For -1453F	
-1453F-F	CGGGGTACCCTCTTTCCACACGGTTCTTTC
-1453F-R	CCGCTCGAGTCTGTGAGGTGGTATCAGCAG
For pcDNA3.1-C/EBPα	
pcDNA3.1-C/EBPα-F	CGCGGATCCATGGAGTCGGCCGACTTCTAC
pcDNA3.1-C/EBPα-R	CCGGAATTCTCACGCGCAGTTGCCCATG
For pLVX_shC/EBPα	
pLVX_shC/EBPα_sense	GATCCGCCGGTACTCGTTGCTGTTCTTCAAGAGAGAACAGCAACGAGTACCGGTTTTTTG
pLVX_shC/EBPα_antisense	AATTCAAAAAACCGGTACTCGTTGCTGTTCTCTCTTGAAGAACAGCAACGAGTACCGGCG
pLVX-NC-sense	GATCCGAGCGTGTAGCTAGCAGAGGTTCAAGAGACCTCTGCTAGCTACACGCTTTTTTTG
pLVX-NC-antisense	AATTCAAAAAAAGCGTGTAGCTAGCAGAGGTCTCTTGAACCTCTGCTAGCTACACGCCG
